# Supplementary figures and images for: Altered kinetics of circulating progenitor cells in cardiopulmonary bypass (CPB) associated vasoplegic patients: A pilot study
Source: PLoS One. 2020 Nov 19;15(11):e0242375. doi: 10.1371/journal.pone.0242375 (PMC7676651; doi:10.1371/journal.pone.0242375)

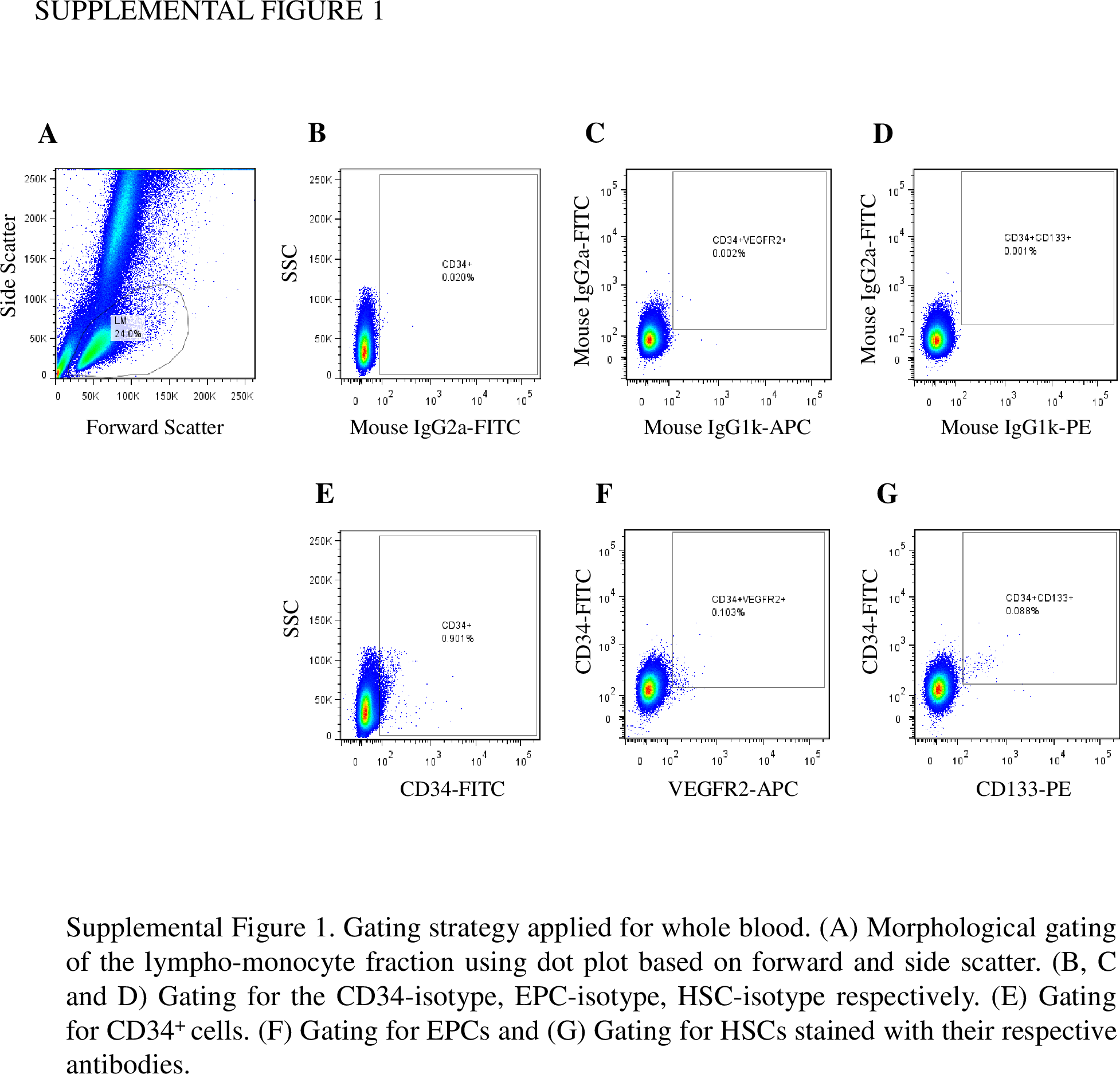

Supplement: S1 Fig — (TIF) [file pone.0242375.s001.tif]

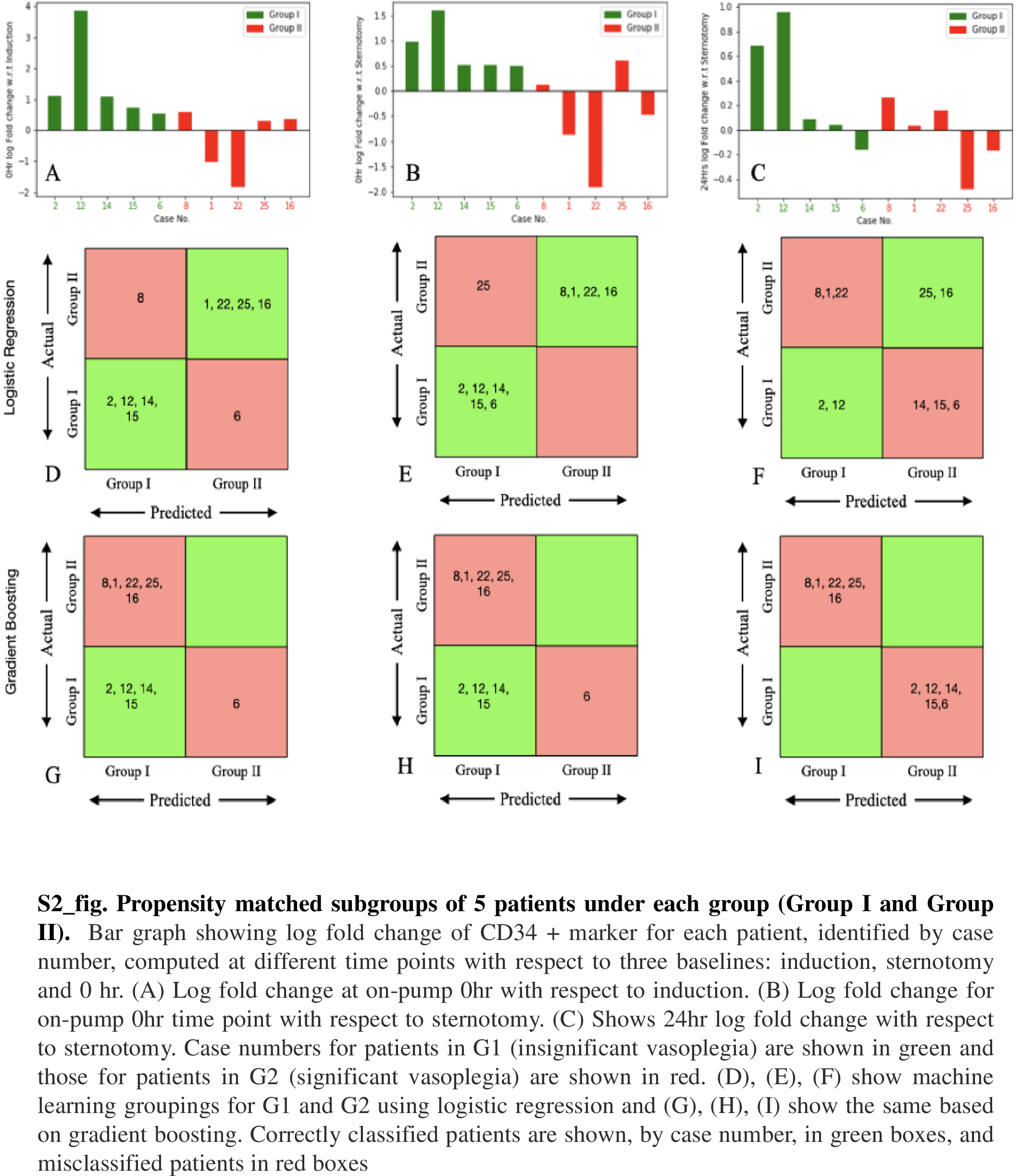

Supplement: S2 Fig — (TIF) [file pone.0242375.s002.tif]

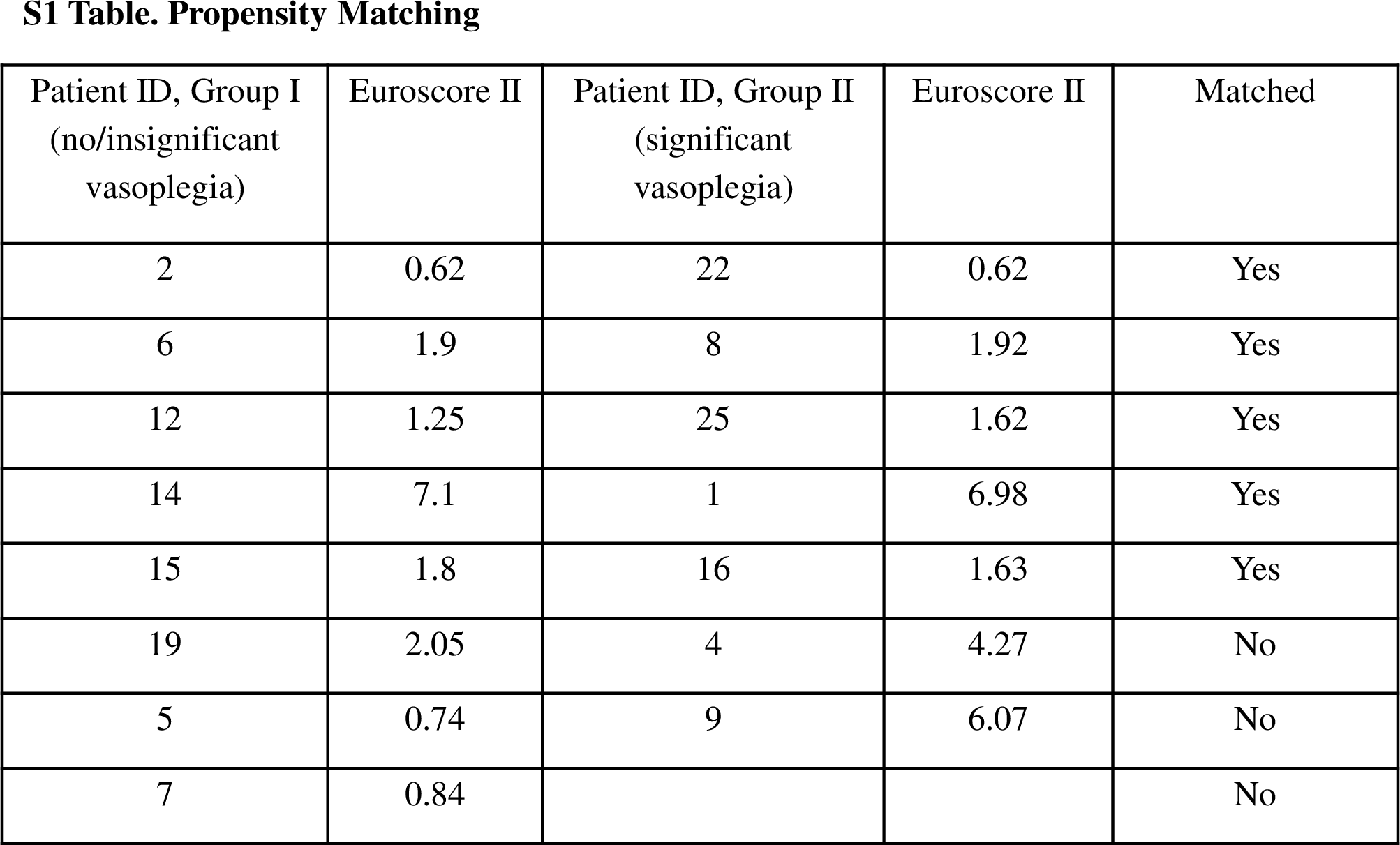

Supplement: S1 Table — (TIF) [file pone.0242375.s003.tif]
