# Supplementary material for: Altered kinetics of circulating progenitor cells in cardiopulmonary bypass (CPB) associated vasoplegic patients: A pilot study
Source: PLoS One. 2020 Nov 19;15(11):e0242375. doi: 10.1371/journal.pone.0242375 (PMC7676651; doi:10.1371/journal.pone.0242375)
Supplement: Attachment — Submitted filename: Response to Reviewers.docx [file pone.0242375.s005.docx]

**Response to Reviewers**

**Editor comment:** A thorough grammar revision is needed.

**Ans:** As per the suggestion by the editor, we have made a thorough grammar revision for the revised manuscript.

**Reviewer #1**

**Query #1:** Thank you for revising your manuscript.

I do very much appreciate the fact that a lot of the data that I requested was added to the manuscript. I think when you talk about vasoplegia, it is important to also have a good clinical picture for such a picture. With regards to this, I think I would like to see more about what to do with this information in a more clinical setting? Do we prophylactically treat these patients who are predisposed to vasoplegia? Please add something short regarding what to do with your information.

Also, please review grammar and English syntax.

I commend the authors on an interesting study and a well written paper.

**Ans**: We thank the reviewer for the comments. We have now included a short summary regarding the findings and perspective of the current pilot study in the discussion section of the revised manuscript.

**Query #2:** The authors correctly addressed my comments and those of the other reviewer. The reason of enrolling such a small population of patients is now better outlined and is reasonable. In my opinion, the pilot nature of the study must be clear from the very title of the paper, so my suggestion is to add "a pilot study" to the title.

**Ans:** We thank the reviewer for the comment. As suggested by the reviewer we have changed the title from ‘Altered kinetics of circulating progenitor cells in cardiopulmonary bypass (CPB) associated vasoplegic patients: An observational study’ to ‘Altered kinetics of circulating progenitor cells in cardiopulmonary bypass (CPB) associated vasoplegic patients: A pilot study’.
